# Co-Operation Schemes

**Published:** 1921-06-18

**Authors:** 


### CO-OPERATION SCHEMES.

Co-operation schemes are already being tried in various localities. In Wiltshire, and again in Dorset, a County Hospitals Association has been formed. In the Dorset scheme the hospitals, we understand, are grouped round Poole, Dorchester, and Weymouth as the chief hospital centres. Each hospital is to have its recognised area, and a *local* association (1) to collect funds by general subscriptions and weekly contributions, and (2) to purchase hospital tickets for distribution at a price approximately to the cost of treatment. The central association will buy all foodstuffs and equipment; establish a common admission rate; form a central fund for special expenditure and audit the accounts of every hospital belonging to it. The plan is to regulate expenditure by income, and to relieve the separate hospitals from the business of collecting funds. Dr. J. Macpherson Lawrie, of the Princess Christian Hospital, Weymouth, has put it, "all direct payment to hospitals would cease." Weekly subscribers to the local associations will be treated free.

A country hospital association with somewhat similar objects has been formed also in Wiltshire, but the Dorset scheme we understand to be the earlier of the two. In the Wiltshire plan co-operative buying is reported to be one of the principal objects, which, of course, do not differ in principle from those engaging attention elsewhere. It is important to inquire, however, what relation these County Hospital associations bear or will bear to the newly formed Regional Committees of the British Hospitals Association, a development which we hoped would prove one of the most useful ever started by that body. Lord Cave's Committee makes recommendation for associations of a similar kind; and there seems a risk of overlapping in the very co-operative schemes which were devised to remedy this defect. It is important that a common area should be decided on. If the county is chosen as the unit, then the county association and the regional committee of the British Hospitals Association should know in what relation they are to stand. For the county associations suggested in the report of the Cave Commission might be the basis of a National Association with which the Government could deal. What is to be the relation of such a body to the British Hospitals Association?

